# Race in public health dentistry: a critical review of the literature

**DOI:** 10.11606/s1518-8787.2022056004173

**Published:** 2022-06-20

**Authors:** Isabela Reginaldo, Isabelle Aparecida Monteiro Fernandes, Giulia Nicoladeli Nuernberg, João Luiz Bastos

**Affiliations:** I Universidade Federal de Santa Catarina Departamento de Saúde Pública Florianópolis SC Brasil Universidade Federal de Santa Catarina. Departamento de Saúde Pública. Florianópolis, SC, Brasil; II Universidade de São Paulo Instituto de Ciências Biomédicas São Paulo SP Brasil Universidade de São Paulo. Instituto de Ciências Biomédicas. São Paulo, SP, Brasil

**Keywords:** Ethnicity and Health, Race Factors, Ethnic Inequality, Social Stigma, Oral Health, Review

## Abstract

**OBJECTIVE:**

To carry out a critical review of the literature on the use of race, color, and ethnicity in the field of public health dentistry.

**METHODS:**

A literature search was conducted in MEDLINE via PubMed for articles published between 2014 and 2019. Using a data extraction form, we collected information on (1) bibliographic characteristics of the selected papers; (2) race, color, and ethnicity of the study participants and their sociodemographic profiles; and (3) the extent to which the original publications followed the recommendations by Kaplan and Bennett (2003) on the use of race, color, or ethnicity in biomedical research.

**RESULTS:**

Our initial search identified 2,032 articles, 53 of which were selected for full-text examination and assessment following pre-established eligibility criteria. Around 60% (n = 32) of the included studies did not justify the use of race, color, or ethnicity in their analyses, and 9% (n = 5) took these variables as indicators of the participants’ genetic makeup. On the other hand, 68% (n = 36) of the reviewed papers considered race, color, and ethnicity as risk markers – not risk factors – for adverse oral health outcomes, whereas 80% (n = 42) adjusted racial/ethnic inequities for a range of socioeconomic and demographic factors in statistical models. Only one study (2%) explicitly took race, color, or ethnicity as a contextually dependent dimension of the participants’ identities.

**CONCLUSION:**

Our findings indicate that research on oral health inequities is often based on reductionist and stigmatizing conceptions of race, color, or ethnicity. Such harmful misconceptions should be replaced with anti-racist narratives in order to effectively address racial oral health inequities.

## INTRODUCTION

Surrounded by controversy and political-ideological struggles, race, color, and ethnicity have been used by dental researchers for over 150 years^1,2^. Many publications from the subfields of orthodontics, periodontics, and public health dentistry, among others, have considered these categories and their relationships with the development of orofacial structures^3^. These discussions are not restricted to orofacial morphology, and also concern the origins of oral health inequities. One of the earliest accounts of racial oral health inequities indicated that “Whites” from Central Europe had more caries than “Blacks” living in South Africa^6^. Recently and especially from the mid-twentieth century onwards, the literature on racial oral health inequities has documented an opposite pattern by showing that racially marginalized groups often have higher frequencies of oral diseases. Studies have also shown that racial gaps in oral health are large, persist over time, and are sometimes partially explained by individual-level indicators of socioeconomic position, including education and income^1,2^.

Amid this discussion, scientists, public health authorities, and health care professionals have come up with several interventions to address racial oral health inequities. Fluoridation of public water supplies, implementation of culturally-sensitive oral health care models, increased access to oral health services, and individualized counseling have often been proposed to address racial oral health inequities^1,7^. Although some of these measures are important and have positive impacts, two recent reviews of the literature identified fundamental limitations in this particular field of knowledge^1,2^: (1) Discussions about the origins of racial oral health inequities often ignore important concepts, such as *systemic racism*; and (2) The use of race, color, and ethnicity in oral health research usually disregards scholarly perspectives from the Humanities and Social Sciences, which are essential to address related inequities.

To the best of our knowledge, only one previous study critically assessed the use of race, color, and ethnicity in the dental public health literature^8^. Though this review indicated that original studies often lacked a systematized procedure for classifying participants and reporting race-related data, it had a number of key limitations, which are worth mentioning here: (1) it was restricted to articles published in *Community Dentistry and Oral Epidemiology* or *Journal of Public Health Dentistry*; (2) non-U.S. populations were excluded from the analysis; and (3) the period analyzed spanned from 2004 to 2009. Most importantly, Susarla et al.^8^ (2014) did not follow any pre-established and agreed-upon criteria to assess the original 140 studies identified. Our study therefore updates and expands upon the previous review by both including original publications from all over the world and assessing the extent to which researchers follow a set of widely accepted recommendations^9^ – detailed below – on the use of race, color, or ethnicity in biomedical publications.

This study critically reviewed the scientific literature on oral health inequities, focusing on articles that used race, color, or ethnicity in their analyses. The limitations of this literature were pointed out, and specific recommendations were made for an ethically-informed and politically-engaged use of these terms in oral health research. By doing so, we assume that only by adopting a critical perspective on the use of these classificatory systems may we effectively mitigate oral health inequities. Throughout this article, we consider racial oral health inequities as the unfair, avoidable, and excessive burden of oral diseases that racially marginalized populations often bear, relative to socially dominant groups^1,2,7^.

## METHODS

We identified dental publications using race, color, or ethnicity in their analyses without imposing limits, such as gender or age, study location, and publication language to the search query. Only articles indexed in the MEDLINE database (via PubMed, https://pubmed.ncbi.nlm.nih.gov/) were considered for review. The search terms used were: (“dentistry” [MeSH] OR “dental health services” [MeSH] OR “oral health” [MeSH]) AND (“skin pigmentation” [MeSH] OR “skin color” [TiAb] OR “race*” [TiAb] OR “racial group” [TiAb] OR “minority groups” [MeSH] OR “population groups” [MeSH] OR “health status disparities” [MeSH] OR “healthcare disparities” [MeSH] OR “racial relations” [MeSH] OR “social discrimination” [MeSH] OR “prejudice” [MeSH] OR “socioeconomic factors” [MeSH]). To update and expand upon the previous work by Susarla et al.^8^ (2014), which focused on publications from 2004 to 2009, we assessed papers published ten years later, i.e., from 2014 to 2019. This period was deemed sufficient for us to potentially document significant changes in the literature under analysis.

To be included in the review, studies should: (1) assess tangible oral health outcomes; (2) use race, color, or ethnicity in their analysis; and (3) examine the relationships between race, color, and ethnicity and the frequency of adverse oral health conditions, using these characteristics for group comparison. Studies that did not meet these inclusion criteria were excluded. In addition, qualitative investigations, editorials, literature reviews, and papers restricted to only one race, color, or ethnic group were not considered for review. A form was specifically developed to extract relevant data from the selected studies, including year of publication, country and institution of origin, journal name, sample size, methods of racial ascription, gender, age, socioeconomic status, and oral health outcomes assessed. We also determined whether original papers followed a series of widely recognized recommendations on the use of race, color, or ethnicity in biomedical research. For this purpose, Kaplan and Bennett’s^9^ (2003) publication was taken as reference. The authors’ propositions, which are detailed below, were included as items in the aforementioned data extraction form, following a similar procedure of a previous literature review^10^.

Firstly, we determined whether race, color, or ethnicity had a central or secondary role in the selected studies. These characteristics were considered of primary importance when they were analyzed alone or with other variables, such as income or education, to determine the distribution of a given oral health outcome. They were considered secondary whenever they were used as a mere classificatory system to describe the studied sample. Other aspects were analyzed as well, including: whether the concept underlying the use of race, color, or ethnicity was defined and if such use was justified; whether race, color, or ethnicity were interpreted as risk factors or risk markers – a risk factor being one major cause of the oral health outcome, and a risk marker, any predictor of the outcome under study that is not causally related to it; whether the study considered other socioeconomic factors (e.g. income, education etc.) to interpret racial oral health inequities, and whether race-, color-, or ethnicity-based inequities were adjusted for these factors in statistical models. We also examined whether authors explicitly recognized the fluid and contextual nature of race, color, and ethnicity in their studies.

The first author extracted data from the selected publications and was supervised by another independent member of the research team, who is the last author. The supervisor double-checked some of the data collected from the original studies to detect typos and problems of interpretation. Data were extracted from the text and tables of the articles identified; whenever this was not possible, study authors were contacted by e-mail for missing or additional information. Publications based on data from the same study were included only once in the review synthesis. The data collected were typed using EpiData version 3.1, with automated controls for consistency and range. Statistical analysis was performed using EpiData Analysis version v.2.2.2.182 to estimate absolute and relative frequencies of each of the items mentioned above in contingency tables.

## RESULTS

We initially identified 2,032 papers with our search query. After checking which papers met the inclusion criteria, 53 scientific articles were selected for analysis – the full list of papers is available as a Supplementary File^a^. Table 1 displays some bibliographic information related to these publications. Nine institutions from Australia, Brazil, Canada, United States of America (USA), England, Norway, and New Zealand stood out with most of the published articles. Table 1 also reveals an absolute growth in the number of published of articles between 2014 and 2018, followed by a slight decrease between 2018 and 2019. Notably, the first authors of 19 out of the 53 reviewed studies were from Brazil. Among the 29 outlets in which the selected articles were published, eight journals were from England, Brazil, or the USA and addressed topics besides oral health, including medicine and public health.

**Table 1:** Bibliographic characteristics of the reviewed studies (n = 53). PubMed, 2014–2019.

Bibliographic characteristics	n	%
First authors’ institutional affiliation		
King’s College	4	7.6
The University of Adelaide	3	5.7
*Universidade de Campinas*	2	3.8
*Universidade Federal de Minas Gerais*	2	3.8
*Universidade Federal do Rio Grande do Sul*	2	3.8
University of Bergen	2	3.8
University of Canterbury	2	3.8
University of Toronto	2	3.8
University of Washington	2	3.8
Remaining institutions	32	60.1
Year of publication		
2014	2	3.8
2015	11	20.8
2016	11	20.8
2017	11	20.8
2018	13	24.5
2019	5	9.4
First authors’ country of origin		
Brazil	19	35.8
United Kingdom	7	13.2
United States of America	6	11.3
Australia	4	7.5
Canada	3	5.7
England	2	3.8
Norway	2	3.8
New Zealand	2	3.8
Netherlands	2	3.8
Denmark	1	1.9
Greece	1	1.9
Israel	1	1.9
Macedonia	1	1.9
Mexico	1	1.9
Switzerland	1	1.9
Journal of publication		
BMC Oral Health	9	17.0
Community Dental Health	4	7.5
PloS One	4	7.5
*Ciência & Saúde Coletiva*	3	5.7
Community Dentistry and Oral Epidemiology	3	5.7
International Dental Journal	3	5.7
Quality of Life Research	3	5.7
*Revista de Saúde Pública*	3	5.7
Remaining journals	21	39.5

Table 2 shows data on the racial/ethnic identification and the sociodemographic characteristics of study participants according to the importance of race, color, and ethnicity for their analyses. Out of the 53 reviewed studies, only nine (17.0%) explicitly mentioned the concept of race, color, or ethnicity used. About 59% (n = 31) of the studies were based on secondary data and therefore did not carry out the racial/ethnic classification of their participants directly, mostly relying on a third party. However, this percentage was slightly higher (64%; n = 7) for publications in which racial/ethnic identification was a secondary finding of the study. Table 2 also shows that 51% of the articles relied on self-classified race, color, and ethnicity, and roughly half of them (45.3%; n = 24) provided a thorough description of the classification process. Around 27% of the studies in which racial/ethnic variables were secondary described how participants were classified regarding race, color, or ethnicity. About 70% (n = 37) of the 53 studies did not clarify whether racial/ethnic information was collected by open-ended or closed-format questions. Most of the 11 studies in which race, color, or ethnicity had a minor role in data analysis and interpretation did not describe how these characteristics were collected (91%; n = 10). The sociodemographic variables most frequently collected in addition to race, color, or ethnicity were gender and socioeconomic status. Few studies (26.4%; n = 14) assessed the participants’ occupation.

**Table 2:** Racial/ethnic and other sociodemographic characteristics of studied participants according to whether race, color, and ethnicity were the primary focus of the reviewed paper (n = 53). PubMed, 2019.

Characteristics	All reviewed papers	Papers which mainly focused on race, color, or ethnicity in the analysis	Papers in which race, color, or ethnicity were secondary to the analysis
	n (%)	n (%)	n (%)
Papers describing the concept of race, color, or ethnicity	9 (17.0)	7 (16.7)	2 (18.2)
Type of data used for racial/ethnic classification			
Primary source	20 (37.7)	16 (38.1)	4 (36.4)
Secondary source	31 (58.8)	24 (57.1)	7 (63.6)
Both primary and secondary sources	2 (3.8)	2 (4.8)	-
Method of racial/ethnic classification			
Self-classification	27 (50.9)	24 (57.1)	3 (27.3)
Hetero-classification	2 (3.8)	2 (4.8)	-
Both self-classification and hetero-classification	-	-	-
Unknown	24 (45.3)	16 (38.1)	8 (72.7)
Use of closed or open-ended items for racial/ethnic classification			
Open-ended item	4 (7.5)	4 (9.5)	-
Closed item	12 (22.6)	11 (26.2)	1 (9.1)
Both closed and open-ended items	-	-	-
Unknown	37 (69.8)	27 (64.3)	10 (90.9)
Sociodemographic characteristics collected besides race, color, or ethnicity			
Sex/gender	46 (86.8)	37 (88.1)	9 (81.8)
Education	34 (64.2)	25 (59.5)	9 (81.8)
Socioeconomic status	41 (77.4)	32 (76.2)	9 (81.8)
Occupation	14 (26.4)	11 (26.2)	3 (27.3)
Total	53 (100.0)	42 (79.2)	11 (20.8)

Table 3 shows the extent to which papers followed the recommendations by Kaplan and Bennett^9^ (2003). Around 60% (n = 32) of the studies did not justify the use of race, color, or ethnicity in their analyses. Five (9.4%) articles used racial/ethnic classification to measure genetic variation, and 36 (67.9%) considered race, color, and ethnicity as risk markers for adverse oral health outcomes. Moreover, racial/ethnic classification was considered alongside other sociodemographic factors in 93% (n = 49) of the articles. Around 80% (n = 42) of the studies adjusted racial/ethnic inequities for other factors in a range of statistical models. Only one (1.9%) out of the 53 articles explicitly considered race, color, and ethnicity as a context-dependent and fluid dimension of the participants’ identity.

**Table 3:** Adherence to Kaplan and Bennett’s (2003) recommendations on the use of race, color, or ethnicity by the reviewed studies (n = 53). Results were stratified by the importance of race, color, or ethnicity for the studies’ analysis and interpretation. PubMed, 2019.

Kaplan and Bennett’s (2003) recommendations	All reviewed papers	Papers which mainly focused on race, color, or ethnicity in the analysis	Papers in which race, color, or ethnicity were secondary to the analysis
	n (%)	n (%)	n (%)
Justified using race, color, or ethnicity	21 (39.6)	16 (38.1)	5 (45.5)
Described how participants were classified according to race, color, or ethnicity	31 (58.5)	28 (66.7)	3 (27.3)
Considered racial/ethnic classification as a proxy for genetic variation	5 (9.4)	5 (11.9)	-
Considered race, color, or ethnicity as a risk factor for adverse oral health	1 (1.9)	1 (2.4)	-
Considered race, color, or ethnicity as a risk marker for adverse oral health	36 (67.9)	30 (71.4)	6 (54.5)
Considered race, color, or ethnicity as a either a risk factor or a risk marker for adverse oral health	2 (3.8)	2 (4.8)	-
Interpreted racial/ethnic inequities in oral health according to contextual specificities and other relevant factors	49 (92.5)	39 (92.9)	10 (90.9)
Adjusted racial/ethnic inequities in oral health for relevant factors in statistical models	42 (79.2)	36 (85.7)	6 (54.5)
Considered racial/ethnic classification as fluid and contextually dependent	1 (1.9)	1 (2.4)	-
Total	53 (100.0)	42 (79.2)	11 (20.8)

## DISCUSSION

By updating and expanding upon Susarla et al.’s^8^ (2014) work, this review showed an absolute increase in the number of dental studies that address race, color, or ethnicity. We also showed that most of the reviewed articles did not follow the recommendations by Kaplan and Bennett^9^ (2003), which were published almost 20 years ago. A comparison between our findings and those of Susarla et al.^8^ (2014) indicates that the percentage of articles which justify the use of race, color, or ethnicity in their analyses has decreased from 50% for papers published between 2004–2009 to around 40% for articles from 2014–2019. Moreover, methods for race, color, or ethnicity assessment were more likely to be omitted in the most recent period. The omission percentage reached 59% in our analysis and 15% in Susarla et al.’s ^8^ (2014) review. However, these comparisons should be done carefully since the original studies were identified, selected, and assessed according to different methods in each review. We thus conclude that the oral health literature has a persistent problem regarding the critical and ethically-responsible use of race, color, or ethnicity in the analyses. Below, we revisit some major findings of our study and provide interpretations that may help advance racial oral health equity. We conclude with some practical recommendations to address the problems identified.

As previously shown, only 17% (n = 9) of the reviewed papers described the concept underlying race, color, or ethnicity, and around 60% (n = 31) relied on secondary data to classify study participants. Historical and social specificities have often been overlooked in the interpretation of racial oral health inequities. Since race, color, and ethnicity are socially- and historically-determined analytical categories, they must be problematized within the corresponding socio-historical context to interpret findings without stigmatizing or perpetuating racial oral health inequities^1,7,11^. About 60% (n = 32) of the studies did not specify the main reason for using racial/ethnic variables in the analysis. According to Kaplan and Bennett^9^ (2003), the reasons for using these variables in biomedical research must be clearly stated upfront to spot and avoid harmful conceptions when developing strategies against racial oral health inequities. Harmful conceptions include those that associate the problem with *independent* individual-level risk factors and disregard racial oral health inequities as the center of intersecting systems of oppression which marginalize groups across multiple life domains^7,b^.

Around 50% (n = 24) of the articles did not describe the process of racial/ethnic classification. This lack of information is concerning, since it prevents readers from understanding how researchers have handled the indeterminacy, subjectivity, and contextual dependency of race, color, and ethnicity. For instance, ignorance of whether race was self- or interviewer-classified suggests that several authors often view it as a fixed characteristic of study participants^1^. Indeed, only one article explicitly considered race, color, and ethnicity as fluid and context-dependent dimensions of the participants’ identity – i.e., it avoided considering racial/ethnic classification as a fixed characteristic by discussing how it may change over time and under different circumstances, depending on a number of temporal, geographic, procedural, and sampling factors^12^. Previous public health studies have already shown how contextually-dependent race can be. In southern Brazil, for example, the participants’ race largely depended on the age, gender, and race of the interviewer: older male respondents were more likely to self-classify as white when approached by younger, black female interviewers^13^.

Most of the publications reviewed (70%; n = 37) also did not mention how racial/ethnic categories were selected by or presented to study participants; thus, the range of available options was unclear. If open-ended questions were used to collect data on race, color, or ethnicity, for example, participants could have more flexibility to fit into the category with which they identify and we could better understand how oral health researchers manage the variability that underlies racial/ethnic classification, particularly when participants must be categorized into a few groups for subsequent statistical analysis^8^. Since some racial/ethnic categorizations collapse groups that are both internally and externally heterogeneous^10^, open-ended questions could better show how estimating adverse oral health conditions among groups based on race, color, or ethnicity is problematic. Without detailed information on the classification process, researchers may unintentionally convey the idea that race/ethnicity can be easily ascribed and that it does not emerge from a negotiation process between the research team and the study participants^13^.

Out of the 53 reviewed articles, only five (9.4%) used race, color, and ethnicity to measure genetic variation and most considered these variables as risk markers, not risk factors, for adverse oral health outcomes. Such findings indicate that some authors still adopt questionable and unethical conceptualizations of race, color, and ethnicity. Our findings also reveal that other relevant factors are often included in the analysis of racial oral health inequities. Specifically, the analytical categories most frequently linked with race, color, and ethnicity were gender and socioeconomic status. The latter analytical category was so frequently associated with racial/ethnic variables that, in some studies, authors even assumed that racial oral health inequities would be completely eliminated by simply reducing socioeconomic gaps between racial/ethnic groups. These publications seemed to adopt a narrow conception of racism, considering it solely as an individual-level phenomenon that does not work *with* and *through* other systems of oppression^1,2,14,15^. Social science scholars have long argued that, together with sexism and classism, racism is a multi-level system that marginalizes racial, color, or ethnic groups by means of economic and noneconomic pathways^14,16^. Oral health researchers have been increasingly drawing from such conceptions^17^, but there is still much room for improvement. Intersectionality and other more elaborate analytical perspectives should thus be clearly articulated with oral health data. Future literature reviews should address the adoption of an intersectionality perspective in oral health inequities research, contributing to the field of general health inequities research^20,21^.

Around 80% (n = 42) of the studies adjusted racial/ethnic inequities for other factors in a range of statistical models. Since these inequities were often associated with socioeconomic disparities among groups, racism and discrimination were rarely considered in the interpretation of findings. Although we must recognize socioeconomic differences across racial/ethnic groups, researchers should also consider that race reflects a multitude of other factors besides income or education. To improve further work on oral health inequities, we thus make the following recommendations, which are in accordance with previous reviews^8,10^ and recent guidelines^22^ on the use of race, color, or ethnicity in medical and science journals:

1. When approaching race, color, or ethnicity in oral health studies, a theoretical or empirical link with racism should be clearly established upfront;
2. Reviewers and editors of scientific articles should ask authors to follow established recommendations^9,10,22^ on the use of these analytical categories to avoid implicit messages of racial hierarchy (organizing racial/ethnic categories in tables and figures in alphabetical order is an example of good practice^22^); and
3. The curriculum of dental schools should be reviewed regarding its sensitivity to racism-related issues and other intersecting forms of injustice, their impacts on population patterns of oral health, and their persistent inequities.

Challenging as they may seem, these recommendations could be more easily followed by establishing partnerships between oral health scholars and researchers from other fields of knowledge, enforcing guidelines for race, color, or ethnicity use across all dental journals, and reforming the curriculum of dental schools considering the oral health needs of racially marginalized populations.

## Footnotes

a Supplementary file available from: https://www.dropbox.com/sh/l0713e7qfmdhfpn/AABytwofk2PuLubYmn_0Q-19a?dl=0.&preview=2021-11-22-Supplementary-file.docx

b Fleming E, Bastos JL, Jamieson L, Celeste RK, Raskin SE, Gomaa N, et al. Inequalities, racism, and discrimination in oral health: a global perspective. Community Dent Oral Epidemiol. 2021;49. Under review.
